# The impact of resistance training on the cognitive health of older adults: a narrative review

**DOI:** 10.3389/fragi.2026.1872457

**Published:** 2026-07-30

**Authors:** Marie Pullerits, Ivi Vaher

**Affiliations:** Physiotherapy and Environmental Health Department, Tartu Applied Health Sciences University, Tartu, Estonia

**Keywords:** ageing, cognitive functionality, cognitive health of older adults, executive brain functions, older adults, elderly training, resistance training

## Abstract

**Background:**

Population aging is associated with an increased risk of cognitive decline, highlighting the need for effective and accessible interventions to support cognitive health in older adults. Resistance training has emerged as a promising non-pharmacological strategy; however, the role of training intensity in optimizing cognitive outcomes remains unclear.

**Methods:**

This narrative review synthesizes 28 peer-reviewed publications, representing 24 independent study samples, published between 2014 and 2024 that examined the effects of resistance training intensity on cognitive function and related physiological markers in older adults.

**Results:**

Evidence indicates that low-, moderate-, and high-intensity resistance training can improve cognitive performance and markers of overall brain and physiological health, including neurotrophic factors, inflammation, and cerebral blood flow. Reported benefits include improvements in executive function, memory, processing speed, and spatial abilities. Moderate-intensity resistance training (approximately 60%–79% of one-repetition maximum) showed the most consistent benefits across studies, though direct intensity comparisons remain limited.

**Conclusions:**

Resistance training, particularly at moderate intensity, appears to be a feasible and effective strategy for supporting cognitive health in older adults, although further research is needed to determine optimal training prescriptions and long-term effects.

## 1 Introduction

Aging is a natural and highly individual process characterized by gradual declines in physical and cognitive capacities, influenced by genetic background and lifestyle factors. In developed countries, older adulthood is commonly defined as age 60 or 65 years and above, a demographic that is rapidly expanding worldwide (United Nations, 2026). Age-related degenerative processes are associated with conditions such as dynapenia, dementia, and general cognitive decline, which pose major challenges for health systems and quality of life (Smolarek et al., 2016). Importantly, cognitive decline does not occur only in the context of overt neurological disease. Mild cognitive impairment (MCI) represents a transitional state between normal aging and dementia–particularly Alzheimer’s disease–and constitutes a critical window for preventive intervention (Hong et al., 2018).

Cognitive function encompasses both crystallized abilities, reflecting accumulated knowledge, and fluid abilities, which involve reasoning, problem-solving, and executive control in novel situations. While crystallized cognition is relatively preserved with age, fluid cognitive abilities typically begin to decline after the age of 60, manifesting as impairments in memory, attention, cognitive flexibility, inhibition, and planning (Coelho-Júnior et al., 2020; de Almeida et al., 2021). Deficits in these domains are not only linked to reduced independence but may also predict future declines in mobility and functional capacity (Best et al., 2015). Consequently, identifying effective strategies to slow cognitive deterioration in older adults is a major public health priority (Hsieh et al., 2016).

Parallel to cognitive aging, advancing age is accompanied by progressive losses in muscle mass and strength, with strength declining more rapidly than muscle mass, particularly under conditions of physical inactivity (Chen et al., 2017). Regular physical activity is a key modifiable lifestyle factor that supports neural adaptation, preserves muscle function, reduces fall risk, and favorably influences neurotrophic and inflammatory processes relevant to cognitive health (Bolandzadeh et al., 2015; Arazi et al., 2021). Compared with pharmacological approaches, physical activity represents a low-cost, accessible, and low-risk intervention with systemic benefits and minimal adverse effects (Hong et al., 2018).

Research on exercise and cognition in older adults has predominantly focused on aerobic or multicomponent training interventions (Tsai et al., 2019). Resistance training, defined as exercise involving muscle contraction against external load, is well established as an effective countermeasure to sarcopenia and related metabolic dysfunctions, which are themselves associated with brain atrophy and cognitive decline (Chen et al., 2017; Herold et al., 2019). Resistance training in older adults has so far been relatively underexplored from a research perspective in the context of cognitive health. Long-term resistance training has been shown to improve executive functions of the brain, such as selective attention, conflict resolution, task switching, working memory, and inhibitory processes (Naderi et al., 2019). A review article by Marston et al. (Marston et al., 2019) indicates that combining resistance training aimed at muscle hypertrophy or strength endurance with aerobic exercise provides greater benefits for cognitive outcomes than aerobic exercise alone. Resistance training also can significantly contribute to the prevention of neurodegenerative diseases as well as to the maintenance, development, and recovery of brain activities through specific neurochemical adaptations induced by the training (Bossers et al., 2015). However, resistance training independently remains underrepresented in cognitive aging research, and findings regarding the optimal training intensity for cognitive benefits are inconsistent.

Recent meta-analytic evidence has begun to synthesize the growing body of research in this area. Wu and Huang (Wu and Huang, 2025) demonstrated significant improvements in global cognition, working memory, and verbal learning following resistance training interventions in older adults, although effects were not uniform across all cognitive domains. Zhang et al. (Zhang et al., 2025) similarly confirmed that resistance training effectively improves muscle strength and functional performance, while effects on muscle mass remain inconsistent, with outcomes depending on training parameters such as frequency, duration, and degree of individualisation, indicating the need for tailored exercise programmes. Notably, neither meta-analysis focused specifically on the role of training intensity as a moderating variable, nor did they systematically examine the neurobiological mechanisms underlying cognitive adaptation.

This narrative review therefore addresses a remaining gap in the literature by synthesizing evidence across low-, moderate-, and high-intensity resistance training protocols, with particular attention to the neurophysiological pathways through which training intensity may differentially influence cognitive outcomes in older adults.

## 2 Methods

This work is a narrative literature review aimed at synthesizing current evidence on the effects of resistance training of varying intensities on cognitive function and related neurobiological markers in older adults. The review focuses on identifying patterns, consistencies, and divergences in the existing research to inform clinical and preventive practice.

A structured search of peer-reviewed scientific literature was conducted in the electronic databases PubMed, ScienceDirect, and Google Scholar. The search targeted studies published between January 2014 and December 2024 in order to capture evidence from the most recent decade of research on the topic. The following keywords and their combinations were used: strength training, resistance training, elderly training, older adults training, cognitive health, cognitive function, and ageing. Boolean operators (AND/OR) were applied as appropriate to combine the search terms. The reference lists of eligible articles were also manually screened to identify additional relevant publications not captured in the initial database search.

The following search equations were used in each database: PubMed (“strength training” OR “resistance training” OR “elderly training” OR “older adults training”) AND (“cognitive health” OR “cognitive function”) AND “ageing”; ScienceDirect (“strength training” OR “resistance training” OR “elderly training” OR “older adults training”) AND (“cognitive health” OR “cognitive function”) AND “ageing”; Google Scholar (“strength training” OR “resistance training” OR “older adults training” OR “elderly training”) AND (“cognitive function” OR “cognitive health”) AND “ageing”.

A total of 28 articles were ultimately included in the synthesis. Given the retrospective nature of this review, exact numbers of records identified, screened, and excluded at each stage were not systematically documented. Accordingly, the final selection reflects a purposive rather than exhaustive or fully reproducible systematic selection, and this work should therefore be interpreted as a narrative review in which included studies represent a considered, though not exhaustive, appraisal of the field.

Studies were included if they met the following criteria: (1) Published between 2014 and 2024; (2) Available as full-text articles; (3) Included participants classified as older adults (generally ≥60 years), who were otherwise generally healthy but may have presented with mild cognitive impairment (MCI), early-stage Alzheimer’s disease, or sarcopenic obesity; (4) Examined resistance training interventions of any intensity, optionally combined with aerobic and/or cognitive training; (5) Reported outcomes related to cognitive performance, neurophysiological measures, or biochemical markers associated with cognitive function. Articles were excluded if they focused exclusively on non-resistance-based interventions, animal models, or populations not representative of older adults.

From each included study, information was extracted on participant characteristics, resistance training protocols (including intensity, volume, frequency, and duration), cognitive outcome measures, and reported neurobiological or biochemical markers. Findings were synthesized qualitatively and grouped according to resistance training intensity (low, moderate, high) to facilitate comparison across studies.

To appraise the methodological quality of included studies, the Physiotherapy Evidence Database (PEDro) scale was applied to all randomised controlled trials and quasi-experimental studies. The PEDro scale comprises 11 criteria, of which 10 contribute to the total score (maximum 10 points), assessing elements such as random allocation, concealed allocation, blinding, follow-up adequacy, intention-to-treat analysis, and reporting of between-group comparisons. Scores were assigned independently by the first author based on information reported in each study. However, it is acknowledged that three PEDro criteria–subject blinding (item 5), therapist blinding (item 6), and assessor blinding (item 7) – are inherently difficult or impossible to satisfy in exercise intervention research, as participants cannot be blinded to whether they are performing physical exercise, and those delivering the intervention are necessarily aware of its nature. This is a well-recognised limitation of applying the PEDro scale to exercise trials and does not reflect a flaw in study design *per se*. Given that these three items are structurally unachievable in exercise research, the effective maximum attainable PEDro score for the majority of studies included in this review is realistically 7 out of 10, rather than the theoretical maximum of 10. Consequently, PEDro scores reported in this review should be interpreted against this adjusted ceiling, and studies scoring 5–6 out of 10 should be considered methodologically adequate within the practical constraints of exercise science.

During the data extraction process, two instances of overlapping samples were identified among the 28 included publications. Macaulay et al. (Macaulay et al., 2021) and Macaulay et al. (Macaulay et al., 2022) are companion publications from the same single-group pilot trial (n = 20), reporting cognitive and neuroimaging outcomes respectively from identical participants. Additionally, Fiatarone Singh et al. (Fiatarone Singh et al., 2014), Suo et al. (Suo et al., 2016), Mavros et al. (Mavros et al., 2017), and Broadhouse et al. (Broadhouse et al., 2020) all derive from the same randomized controlled trial–the SMART Trial (n = 100 older adults with MCI) – each reporting distinct outcome domains from the same cohort. Where publications shared the same participant sample, they were treated collectively as a single independent source of evidence, identified as such in the summary tables, and interpreted as complementary rather than independently replicating evidence throughout the Results and Discussion.

In the context of resistance training, intensity refers to the percentage of the maximum load that can be lifted for one repetition, i.e., % of 1RM (Garber et al., 2011). Thus, training intensity reflects how close the performance is to the individual’s maximum capacity. Resistance training can be classified by intensity into low-, moderate-, and high-intensity categories. Low-intensity resistance training, aimed at developing muscular endurance, uses light weights (40%–60% of 1RM), a high number of repetitions (12–20), and 2–3 sets (de Almeida et al., 2021). To increase muscle mass and activity, moderate-intensity resistance training aimed at muscular hypertrophy has traditionally been used. In this case, the weight range is 60%–79% of 1RM, the number of repetitions is typically 8–12, and 2–3 sets are performed. High-intensity resistance training (80%–100% of 1RM) is aimed at developing maximal strength. At this intensity, typically 3–4 sets are performed, each consisting of 4–6 repetitions (Macaulay et al., 2022).

This narrative review is based solely on previously published data. No new datasets were generated, and no primary data, materials, or computer code were produced. All data sources are publicly available through the cited publications. As no new human or animal research was conducted, ethical approval was not required.

## 3 Results

The narrative review article includes a total of 28 studies. Of the included studies, nine confirmed the beneficial effects of low-intensity resistance training on cognitive performance or biomarkers (Bossers et al., 2015; Yoon et al., 2017; Yoon et al., 2018; Hong et al., 2018; Sardeli et al., 2018; Naderi et al., 2019; Urzi et al., 2019; Coelho-Júnior et al., 2020; de Almeida et al., 2021), eleven studies (Fragala et al., 2014; Gomes de Souza Vale and Ferreira, 2014; Tsai et al., 2015; Johnson et al., 2016; Hsieh et al., 2016; Smolarek et al., 2016; Chen et al., 2017; Tsai et al., 2017; Arazi et al., 2021; Tsai et al., 2019; Naderi et al., 2019) used moderate-intensity, muscle-hypertrophy-oriented resistance training as the experimental intervention, and nine studies (Fiatarone Singh et al., 2014; Best et al., 2015; Bolandzadeh et al., 2015; Suo et al., 2016; Mavros et al., 2017; Macaulay et al., 2021; Macaulay et al., 2022; Broadhouse et al., 2020; Sheoran et al., 2023) employed high-intensity resistance training as the intervention.

### 3.1 Effects of low-intensity resistance (muscular endurance) training

Low-intensity resistance training aimed at developing muscular endurance uses light weights (50%–65% of 1 repetition-max, 1RM), a high number of repetitions (12–20), 2–3 sets, and short rest periods between sets (20–30 s). In certain clinical conditions, such as in older adults with obesity, low-intensity muscular endurance training may be the only applicable and sustainable form of resistance training (de Almeida et al., 2021).

Low-intensity resistance training has been shown to produce modest improvements in cognitive function in older adults, including executive function, working memory, attention, and reaction time (See Table 1).

**TABLE 1:** Low-intensity resistance training.

Study	Type of study	PEDro score**	Acute effects vs chronic changes	Population (n, age, cognitive status, if specified)	Intervention (intensity, duration, frequency)	Outcome measures	Cognitive effects and/or neurochemical changes from resistance training	Notes/ Special findings
Bossers et al., 2015	Randomized controlled trial (RCT)	6/10	Chronic changes in 9 weeks	Older adults with moderate–severe dementia (n = 109; age 85,5 ± 5.1 year)	Low- to mid-intensity resistance + aerobic training (RPE* 12–15/20); 9 weeks; 2–3×/week	Cognitive function: MMSE, WMS-R, Stroop test, verbal fluency test, Groningen Intelligence Test, trail making test-A	Global cognitive function;, verbal memory ↑; visual memory ↑; executive function ↑	Combined training more effective than aerobic exercise alone
Yoon et al., 2017	RCT	5/10	Chronic changes in 12 and 16 weeks	Older adults (n = 30; 74–79 years)	Low-intensity resistance endurance training with elastic bands; 12 and 16 weeks; 3×/week; fast vs slow tempo	Cognitive function: MMSE, MoCA	MMSE ↑ 21%, MoCA ↑ 33%	Greater improvements with fast-tempo training
Yoon et al., 2018	RCT	5/10	Chronic changes in 16 weeks	Older adults with mild cognitive impairment (n ≈ 45; 69–73 years)	High-speed- low-intensity resistance endurance training with elastic bands; 16 weeks; 3×/week	Cognitive function: MMSE-K, CERAD-K, BNT, REY-15, TMT-A, TMT-B, Digit Span test, FAB	Cognitive function ↑ (memory ↑, processing speed ↑, cognitive flexibility ↑, working memory ↑, executive function ↑)	Higher neuromuscular and attentional demands proposed
Hong et al., 2018	RCT	5/10	Chronic changes in 12 weeks	Older adults with mild cognitive impairment (n = 47, 76–92 years)	Low-intensity resistance endurance training with elastic bands ∼65% 1RM; 12 weeks; 3×/week	EEG, cognitive outcomes; working memory, selective attention, physical fitness	EEG parameters ↑, working memory ↑, attention ↑	Demonstrated neurophysiological adaptations alongside cognitive gains
Sardeli et al., 2018	Crossover Trial†	5/10	Acute effects	Healthy older adults (n ≈ 24; ≥60 years)	Single acute low- (30% 1RM) vs moderate-intensity (80% 1RM) leg press session	Cognitive outcomes: Stroop test	Reaction time ↑	Low-intensity session improved reaction time more than high intensity. Acute effects linked to cardiovascular response and motor learning
Urzi et al., 2019	RCT	5/10	Acute effects	Older female adults (n = 20; age 84 ± 8 years)	Single elastic-band resistance training session (low intensity)	Serum BDNF, IL-8	BDNF ↑, IL-8 ↓	Supports neurotrophic and anti-inflammatory mechanisms
Naderi et al., 2019	Crossover Trial	5/10	Chronic changes in 8–12 weeks	Older adults (n = 40; 60–75 years)	Low-intensity (40% 1 RM) vs moderate-intensity (70% 1 RM) resistance training; 8–12 weeks; 2–3×/week	Executive function: working memory, response inhibition and cognitive flexibility	Both intensities improved cognition and executive function; moderate intensity superior	Moderate intensity had a superior effect on cognition improvements
Coelho-Júnior et al., 2020	RCT	6/10	Chronic changes in 22 weeks	Older female adults (n = 36; 66.8 ± 5.1 yrs)	Low- vs moderate-intensity resistance training; 22 weeks; 2×/week	Global cognitive function: MMSE, TUG-cog, dual task performance; GDS; BDNF	MMSE ↑; short term memory ↑; dual-task performance ↑; depression levels ↓	Moderate intensity training showed larger cognitive gains and added benefits
de Almeida et al., 2021	Counterbalanced Crossover Trial	5/10	Chronic changes in 12 weeks	Obese older adults (n = 31; 63–71 years)	Low- and moderate-intensity resistance training vs functional training; 12 weeks; 3×/week	Executive function: Stroop test, Trail-Making Test	Executive function ↑, inhibition function ↑	Functional training comparable to resistance training

** PEDro, Physiotherapy Evidence Database scale (maximum score 10). Scores reflect independent assessment by the authors based on reported methodological information. Scores of 9–10 = excellent; 6–8 = good; 4–5 = fair; <4 = poor. Blinding of participants (criterion 5) and therapists (criterion 6) was not feasible for exercise intervention studies and scored 0 across all included studies.

* 1RM, One-Repetition Maximum; BDNF, Brain-Derived Neurotrophic Factor; BNTREY-15, Boston Naming Test–Rey 15-item version; CERAD-K, Consortium to Establish a Registry for Alzheimer’s Disease, Korean version; EEG, Electroencephalography; FAB, Frontal Assessment Battery; GDS, Geriatric Depression Scale; IL-8, cytokine Interleukin 8; MMSE, Mini-Mental State Examination; MMSE-K, Mini-Mental State Examination, Korean version; MoCA, Montreal Cognitive Assessment Test; RPE, rate of perceived exertion; TMT-A, Trail Making Test Part A; TMT-B, Trail Making Test Part B; TUG-cog, Timed Up and Go Test, Cognitive version; WMS-R, Wechsler Memory Scale, Revised.

† Crossover design–PEDro, criteria interpreted accordingly; criterion 9 (ITT) not applicable. The 80% 1RM, condition represents a within-study comparator rather than an independent high-intensity intervention; the study’s primary focus is the acute effects of low-intensity exercise and is therefore classified under low-intensity training.

Most studies compared low-intensity protocols with non-exercising controls or stretching/mobility interventions, whereas only a few directly compared low- versus moderate-intensity resistance training (Sardeli et al., 2018; Naderi et al., 2019; Coelho-Júnior et al., 2020; de Almeida et al., 2021). These comparative studies indicate that while low-intensity training is beneficial, moderate-intensity training may confer larger cognitive gains. It should be noted that the majority of low-intensity studies in this review used elastic-band resistance, which provides accommodating rather than constant-load resistance. This limits direct comparison with free-weight or machine-based low-intensity protocols used in other studies.

Acute effects of low-intensity training sessions include increased cerebral blood flow and modulation of the autonomic nervous system. For example, (Sardeli et al., 2018) reported that a single low-intensity leg-press session improved reaction time on the Stroop test more than higher-intensity exercise, likely due to a more dynamic cardiovascular response and facilitation of motor learning. Urzi et al. (2019) found that a single elastic-band session increased Brain-Derived Neurotrophic Factor (BDNF) and decreased Interleukin-8 (IL-8) levels, supporting the role of neurotrophic and anti-inflammatory pathways in cognitive enhancement.

Longer-term interventions further support cognitive benefits. Hong et al. (2018) observed that a 12-week low-intensity elastic-band program (65% of 1 repetition-max (1RM), 15 repetitions per set) improved electroencephalography (EEG) parameters, working memory and selective attention in older adults with mild cognitive impairment. Yoon et al. (Yoon et al., 2017; Yoon et al., 2018) have highlighted the importance of exercise tempo: fast-tempo resistance endurance training led to greater improvements in Mini-Mental State Examination (MMSE) and Montreal Cognitive Assessment (MoCA) scores than slow-tempo protocols, likely due to increased neuromuscular activation, attention demands, and circulatory responses.

Low-intensity resistance training is also effective in populations with metabolic impairments. De Almeida et al. (de Almeida et al., 2021) demonstrated that low- and moderate-intensity resistance training, as well as functional training mimicking daily activities, improved executive function in obese older adults. Additionally, in older adults with moderate to severe dementia, combined low-intensity resistance and aerobic training attenuated cognitive decline more effectively than aerobic training alone (Bossers et al., 2015).

Importantly, the direction of findings was generally consistent across cognitively healthy older adults and those with cognitive impairment. Studies involving older adults with mild cognitive impairment (Hong et al., 2018) and dementia (Bossers et al., 2015) demonstrated improvements or attenuation of cognitive decline comparable to those observed in cognitively healthy populations. Although the magnitude of effects varied between studies, the available evidence suggests that low-intensity resistance training can positively influence cognitive function regardless of baseline cognitive status. These findings are particularly relevant because they indicate that cognitive impairment does not preclude responsiveness to resistance-training interventions.

While low-intensity resistance training is safe, feasible, and suitable for home- or community-based interventions, evidence suggests that moderate-intensity hypertrophy-focused protocols may produce greater cognitive gains, particularly in executive functions and working memory.

### 3.2 Effects of moderate-intensity resistance training aimed at muscular hypertrophy

Moderate-intensity resistance training, typically performed at 60%–79% of one-repetition maximum (1RM) or rated 5–6/10 on perceived exertion scales, has consistently been shown to improve cognitive function in older adults, particularly executive function, working memory, attention, and reaction time (See Table 2).

**TABLE 2:** Moderate-intensity resistance training.

Study	Type of study	PEDro score**	Acute effects vs chronic changes	Population (n, age, cognitive health status)	Intervention (intensity, duration, frequency)	Outcome measures	Cognitive effects and/or neurochemical changes from resistance training	Notes/ Special findings
Fragala et al., 2014	Quasi-experimental pretest-posttest study	4/10	Chronic changes in 6 weeks	Older adults (n = 25; age 70.6 ± 6. yrs)	Moderate-intensity resistance training (OMNI scale ∼5–6/10); 6 weeks; 2×/week	Cognitive function: Spatial awareness, peripheral, visual, motor, and physical reaction times, circulating BNDF	Spatial awareness ↑; visual reaction time ↑; physical reaction time ↑	The lack of BDNF change suggests cognitive improvements may not be mediated by chronic increases in resting BDNF
Gomez de Souza Vale 2014	Controlled pre-post intervention study	4/10	Chronic changes in 20 weeks	Elderly women (n = 24: age 64–74)	Moderate-intensity resistance training OMNI scale ∼5–6/10); 20 weeks; 3×/week	Functional autonomy (GDLAM*), AI, circulating IGF-1	Functional autonomy ↑; AI ↑; IGF-1 ↑	Results suggest a possible link between resistance training, anabolic hormonal response and improved functional independence in elderly women
Tsai et al., 2015	Randomized controlled trial (RCT)	5/10	Chronic changes in 12 months	Healthy elderly men (n = 48; 67–76 years)	Moderate-intensity resistance training; 12 months; 3×/week	Executive function: RT; Cognitive processing speed: ERP components P3a and P3b; GH, IGF-1, serum homocysteine	RT ↓; Cognitive processing speed ↑; IGF-1 ↑; serum homocysteine↓	The increase in serum IGF-1 with exercise was significantly correlated with both faster reactions and better neuroelectric responses
Johnson et al., 2016	Acute experimental study	5/10	Acute effects	Healthy older adults (n = 31, average age 71 years)	Moderate-intensity resistance training (60% 1 RM) vs aerobic training; 4weeks; 1×/week	Executive function: Stroop inhibition test	Executive function ↑	A single bout of exercise (both aerobic and resistance) can acutely enhance executive processing in older adults
Hsieh et al., 2016	Acute experimental study	4/10	Acute effects	Healthy younger men (n = 18, aged 21–25 years) and healthy older men (n = 11, aged 65–67 years)	Single acute moderate-intensity (70% 1RM) resistance training, three sessions	Cognitive function: Sternberg working memory task; RT	Working memory ↑; RT ↓	A single bout of resistance exercise speeded reaction time specifically for some working memory conditions in older adults
Smolarek et al., 2016	RCT	5/10	Chronic changes in 12 weeks	Healthy sedentary elderly women (n = 29; age 65,8 ± 5.7 years)	Moderate-intensity resistance training (60%–75% 1 RM) vs aerobic training; 12 weeks; 3×/week	Cognitive capacity: MoCA	MoCa ↑	A positive association between improvements in lower limb strength and cognitive outcomes
Chen et al., 2017	Multi-arm RCT	5/10	Chronic changes in 8 weeks	Older adults with sarcopenic obesity (n = 60; 65–75 years)	Moderate-intensity resistance training vs aerobic training vs combined training vs control group; 8 weeks; 2×/week	Blood IGF-1	IGF-1 ↑	Only the resistance training group had a significant increase in IGF-1 after 8 weeks, indicating potential anabolic and neurotrophic effects
Tsai et al., 2017	Acute crossover trial	5/10	Acute effects	Older adults with mild cognitive impairment (n = 66; 60–80 years)	Moderate-intensity resistance training vs aerobic training vs control group; single acute training session	Executive function: MMSE; EEG: ERP P3 amplitude and latency; BNDF; IGF-1; VEGF; FGF-2	IGF-1 ↑; BDNF ↑	Results suggest distinct biological pathways depending on training mode
Naderi et al., 2019	Acute crossover trial	5/10	Chronic changes in 12 weeks	Older adults (n = 40; 60–75 years)	Low-intensity (40% 1 RM) vs moderate-intensity (70% 1 RM) resistance training; 8–12 weeks; 2–3×/week	Executive function: working memory, response inhibition and cognitive flexibility	Both intensities improved cognition and executive function; moderate intensity superior	Moderate intensity had a superior effect on cognition improvements
Tsai et al., 2019	RCT	5/10	Chronic changes in 16 weeks	Older adults with amnestic mild cognitive impairment (n = 55; 59–73 years)	Moderate-intensity resistance training (60%–75% 1 RM) vs aerobic training vs control group; 16 weeks; 3×/week	Neurocognitive performance: accuracy rates, reaction times; EEG: ERP P3 amplitudes; neurotrophic factors BDNF, IGF-1 VEGF, FGF-2; inflammatory cytokines TNF-α, IL-1β, IL-6, IL-8, IL-15	Neurocognitive performance ↑; ERP P3 amplitudes ↑; IGF-1 ↑; IL-8 ↓	Aerobic exercise primarily increases BDNF and reduces inflammation, while resistance exercise more strongly elevates IGF-1, indicating that exercise mode differentially influences neurochemical adaptations
Arazi et al., 2021	Acute crossover trial	5/10	Acute effects	Healthy elderly men (n = 30; 60.8 ± 1.8 years)	Moderate-intensity resistance training (65%–70% 1 RM) vs aerobic training vs control group; single acute training session	BDNF, IGF-1, Platelet count	BDNF ↑; IGF-1 ↑; Platelet counts ↑	Results suggest that even a single session of acute moderate-intensity resistance exercise stimulates biomarkers linked with brain plasticity

** PEDro, Physiotherapy Evidence Database scale (maximum score 10). Scores reflect independent assessment by the authors based on reported methodological information. Scores of 9–10 = excellent; 6–8 = good; 4–5 = fair; <4 = poor. Blinding of participants (criterion 5) and therapists (criterion 6) was not feasible for exercise intervention studies and scored 0 across all included studies.

* AI, Autonomy Index; BDNF, Brain-Derived Neurotrophic Factor; EEG, Electroencephalography; ERP, Event-Related Potential; FGF-2, Fibroblast Growth Factor 2; GH, Growth Hormone; GDLAM, Latin American Development Group for Maturity Protocol; IGF-1, Insulin-Like Growth Factor 1; IL-1, Interleukin 1; IL-6, Interleukin 6; IL-8, Interleukin 8; IL-15, Interleukin 15; MMSE, Mini-Mental State Examination; MoCA, Montreal Cognitive Assessment Test; OMNI–OMNI, rating of perceived exertion scale; P3a, P3b–Event-Related Potential Components; RT, Reaction Time; TNF, Tumor Necrosis Factor Alpha; VEGF, Vascular Endothelial Growth Factor.

Interventions typically consisted of two to three sessions per week, lasting 30–60 min, with total program duration ranging from 8 to 24 weeks. Progressive overload protocols, implemented in several studies (Fragala et al., 2014; Tsai et al., 2015; Johnson et al., 2016; Tsai et al., 2019), gradually increased load or volume in response to participants’ strength gains, stimulating both muscular and neuroplastic adaptations.

Acute effects of moderate-intensity resistance training have been additionally documented. Even a single session can transiently enhance cognitive performance, likely through increased cortical arousal, catecholamine release, and upregulation of neurotrophic factors such as Brain-Derived Neurotrophic Factor (BDNF) and Insulin-Like Growth Factor 1 (IGF-1). Hsieh et al. (Hsieh et al., 2016), in a study primarily conducted in young men but including a small older male subgroup (n = 11, aged 65–67), reported that a single whole-body session at 70% 1RM improved working memory in the older subgroup more than in younger participants, particularly on tasks requiring higher executive function. These findings should however be interpreted cautiously given the small size and secondary nature of the older adult sample. Similarly, Johnson et al. (2016) found that short 10- and 30-min moderate-intensity resistance training sessions improved Stroop inhibition performance immediately post-exercise, suggesting that even short moderate-intensity training optimally stimulates attention, arousal, and neural processing. Tsai et al. (Tsai et al., 2017) observed that acute moderate-intensity resistance training increased IGF-1 levels and improved reaction time, although IGF-1 returned to baseline within 20 min, indicating a temporary but measurable neurophysiological response. Nevertheless, moderate-intensity resistance training may regulate the expression of growth factors (e.g., BDNF, IGF-1, and Vascular Endothelial Growth Factor (VEGF)), slow neurodegenerative processes, and reduce long-term dementia risk.

Longer-term interventions demonstrate that repeated moderate-intensity training can produce sustained cognitive and/or neurobiological improvements. Smolarek et al. (Smolarek et al., 2016) reported that 12 weeks of moderate-intensity resistance training in sedentary older women (n = 29, mean age 65.8 ± 5.7 years) led to 58% and 68% gains in upper- and lower-limb strength, accompanied by a 19% improvement in cognitive function as measured by Montreal Cognitive Assessment Test (MoCA). A 20-week cycle of moderate-intensity resistance training was shown to increase IGF-1 levels in older adults in a study by Gomes de Souza Vale and Ferreira (2014) with older women (n = 24, aged 64–74). Higher IGF-1 levels are associated with greater overall brain volume in both cognitively healthy individuals, as well as in those with mild cognitive impairments (Marston et al., 2019). In the study by Tsai et al. (2015), 12 months of moderate-intensity resistance training, performed three times per week, was shown to increase BDNF, GH, and IGF-1 levels and reduce homocysteine in healthy men aged 67–76 (n = 48). These changes suggest that regular resistance training may delay age-related functional decline and help maintain cognitive function, as high homocysteine is a risk factor for cognitive impairment. Overall the findings suggest that improvements in muscular strength may facilitate cognitive enhancement through mechanisms such as increased blood flow, neuromuscular activation, and greater engagement of attention and motor coordination networks.

Several studies have compared low- versus moderate-intensity protocols, demonstrating superior cognitive benefits for moderate-intensity training. Naderi et al. (2019) reported that healthy adults aged 60–75 years performing resistance training at 70% 1RM showed greater improvements in executive functions than those exercising at 40% 1RM, consistent with the cognitive-energetic model: moderate-intensity exercise places higher cognitive demands and produces optimal arousal for information processing. Coelho-Júnior et al. (2020) examined women aged over 60 over a period of 22 weeks and observed that moderate-intensity training improved overall cognitive function (16.2% vs. 14.4%), short-term memory (56.1% vs. 39.3%), and dual-task performance (43.1% vs. 52.7%) more than low-intensity protocols, although no significant changes in BDNF were detected. Collectively, these results suggest that a moderate intensity threshold may be necessary to elicit meaningful cognitive adaptations in older adults.

Moderate-intensity resistance training has also been shown to benefit populations with cognitive or metabolic vulnerabilities. Tsai et al. (2019) reported that older adults with mild amnestic cognitive impairment (n = 55, aged 60–85) improved reaction times, working memory, and blood biomarkers–significantly increased IGF-1 levels and decreased Interleukin-15 (IL-15) levels–after a 4-month moderate-intensity program, suggesting partially distinct neurological mechanisms for cognitive and physical adaptations. Importantly, studies indicate that benefits emerge not only from repeated interventions but also from single sessions, making moderate-intensity training a feasible strategy for both acute and long-term cognitive enhancement.

A notable finding across the moderate-intensity studies is that cognitive improvements were observed in both cognitively healthy older adults and those with mild cognitive impairment. For example, healthy participants demonstrated improvements in executive function, working memory, reaction time, and neurotrophic biomarkers (Smolarek et al., 2016; Naderi et al., 2019; Gomes de Souza Vale and Ferreira, 2014; Tsai et al., 2015), while similar benefits were reported in individuals with mild amnestic cognitive impairment (Tsai et al., 2019). The consistency of these findings across populations suggests that moderate-intensity resistance training may support cognitive function irrespective of baseline cognitive status and may help preserve or partially restore cognitive performance in older adults already experiencing early cognitive decline.

Smolarek et al. (2016), Chen et al. (2017), and Arazi et al. (2021) consistently demonstrate that moderate-intensity resistance training improves physical and neurocognitive-related outcomes in older adults. Smolarek et al. (2016) reported substantial gains in upper and lower limb strength alongside improved cognitive performance after 12 weeks of training. Chen et al. (2017) found significant improvements in body composition, muscle strength, and serum IGF-1 concentrations in older adults with sarcopenic obesity, with benefits maintained 1 month post-intervention. Similarly, Arazi et al. (2021) showed that acute moderate-intensity resistance exercise increased serum BDNF and IGF-1 levels and platelet counts in older men. Collectively, these findings suggest that moderate-intensity resistance training enhances muscular function and upregulates neurotrophic and anabolic factors associated with cognitive health and neuroplasticity in aging populations.

Overall, moderate-intensity (approximately 60%–70% 1RM), hypertrophy-focused resistance training appears particularly effective for improving executive function, reaction time, and working memory in older adults. Beneficial effects have been observed in both cognitively healthy individuals and those with mild cognitive impairment, suggesting broad applicability across different stages of cognitive aging. Effects are observed both acutely and following sustained interventions, with greater benefits than low-intensity protocols.

### 3.3 Effects of high-intensity resistance training

High-intensity resistance training, typically performed at 80%–95% of one-repetition maximum (1RM), targets maximal strength development and may produce unique cognitive benefits through increased neuromuscular activation and strength gains (see Table 3).

**TABLE 3:** High-intensity resistance training.

Study	Type of study	PEDro score**	Acute effects vs chronic changes	Population (n, age, cognitive health status)	Intervention (intensity, duration, frequency)	Outcome measures	Cognitive effects and/or neurochemical changes from resistance training	Notes/ Special findings	Trial affiliations
Fiatarone Singh et al., 2014	Randomized Controlled Trial (RCT)	7/10	Chronic changes in 6 months	Older adults with mild cognitive impairment (n = 100; age 70.1 ± 6.7 years)	High-intensity progressive resistance training vs seated calisthenics vs multidomain cognitive training vs watching videos/quizzes; 6 months; 2–3×/week	Global cognitive function: ADAS-Cog; Functional independence: B-ADL; Executive function	ADAS-Cog ↑; Executive function ↑; B-ADL ↑	Resistance training alone showed independent cognitive benefits beyond the effects of cognitive or combined training. Benefits of resistance training on cognition were maintained up to 18 months	SMART Trial – shared cohort (n = 100)†
Best et al., 2015	RCT	6/10	Chronic changes in 52 weeks	Community-dwelling elderly women (n = 155: age 65–75)	High-intensity resistance training vs balance-and-tone training; 52 weeks; 1–2×/week	Executive function; verbal memory; Cortical white matter lesion volume; Cortical gray matter volume; Hippocampal volume	Executive function ↑; verbal memory ↑; Cortical white-matter atrophy ↓	Twice-weekly resistance training reduced cortical white-matter atrophy over 2 years; cognitive benefits persisted 1 year after the intervention	​
Bolandzadeh et al., 2015	RCT	6/10	Chronic changes in 52 weeks	Community-dwelling elderly women with white matter lesions (n = 54: age 65–75)	High-intensity resistance training vs balance-and-tone training; 52 weeks; 1–2×/week	Executive function; Cortical white matter lesion volume	Cortical white-matter atrophy ↓	The results suggest potential long-term positive effects of high-intensity resistance training on structural changes in the brain	​
Suo et al., 2016	RCT	7/10	Chronic changes in 6 months	Older adults with Mild Cognitive Impairment (n = 100, age 70.1 ± 6.7 years)	High-intensity resistance training vs stretching and seated calisthenics vs cognitive training vs watching educational videos; 6 months; 2×/week	Global cognition: ADAS-Cog; cortical thickness: gray matter volumes: white matter volumes; PC cortical thickness	ADAS-Cog ↑; PC cortical thickness ↑; Cortical white-matter atrophy ↓	High-intensity resistance training uniquely affected structural brain markers implicated in Alzheimer’s progression, suggesting potential disease-modifying effects	SMART Trial – shared cohort (n = 100)†
Mavros et al., 2017	SMART RCT	7/10	Chronic changes in 6 months	Older adults with mild cognitive impairment (n = 100; average age 69,5 years)	High-intensity resistance training (85%–92% 1RM) vs cognitive training vs control group, 6 months, 2–3x/week	Global cognition: ADAS-Cog; VO_2_peak	ADAS-Cog ↑	Cognitive benefits were primarily explained by the improvements in muscle strength, not in aerobic fitness	SMART Trial – shared cohort (n = 100)†
Macaulay et al., 2021	Quasi-experimental pre-post design	4/10	Chronic changes in 12 weeks	Healthy older adults (n = 20; age 69.1 ± 5.8 years)	High-intensity resistance training; 12 weeks; 3×/week	Fluid cognition: executive function, attention, working memory, processing speed; Crystallized cognition	Executive function ↑; attention ↑; working memory ↑; processing speed ↑	Muscular power showed a slightly stronger association with cognition changes in comparison with muscular strength	Companion publication – shared cohort (n = 20)‡
Macaulay et al., 2022	Quasi-experimental study	3/10	Chronic changes in 12 weeks	Healthy older adults (n = 20; age 69.1 ± 5.8 years)	High-intensity resistance training; 12 weeks; 3×/week	Fluid cognition; Resting-state functional brain efficiency; CBF; cortical white-matter volume	Fluid cognition ↑; Resting-state functional brain efficiency ↑; CBF ↑; white-matter atrophy ↓	Small favorable effects on cerebrovascular function suggest plausibility of vascular and neuroplastic mechanisms	Companion publication – shared cohort (n = 20)‡
Broadhouse et al., 2020	SMART RCT	6/10	Chronic changes in 6 months	Older adults with mild cognitive impairment (n = 100; age 69.5 ± 6.6 years)	High-intensity resistance training (80% 1 RM) vs stretching and calisthenics vs cognitive training in combined groups; 6 months; 2–3x/week acute training session	Global cognition: ADAS-Cog; Executive function; Hippocampal subfield volumes; Cortical thickness; Resting-state functional brain connectivity	ADAS-Cog ↑; Executive function ↑; Hippocampal subfield volume atrophy ↓; Short-term cortical thickness ↑; Resting-state functional connectivity ↑	Long-term (18 months post-intervention) cognitive improvements were observed with high-intensity resistance training alone	SMART Trial – shared cohort (n = 100)†
Sheoran et al., 2023	RCT	6/10	Chronic changes in 12 weeks	Healthy older adults (n = 41; 60–80 years)	High-intensity (70%–85% 1 RM) vs control group; 12 weeks; 2–3×/week	Ratios of neurometabolites in three brain regions: Hippocampus, Primary Sensorimotor Cortex, Dorsolateral Prefrontal Cortex	Neurometabolic integrity preserved in the resistance training group	High-intensity resistance training appeared to counteract age-related decreases in neuronal and neurotransmitter markers, particularly in sensorimotor and prefrontal cortices	​

** PEDro, Physiotherapy Evidence Database scale (maximum score 10). Scores reflect independent assessment by the authors based on reported methodological information. Scores of 9–10 = excellent; 6–8 = good; 4–5 = fair; <4 = poor. Blinding of participants (criterion 5) and therapists (criterion 6) was not feasible for exercise intervention studies and scored 0 across all included studies.

* ADAS-Cog–Alzheimer’s Disease Assessment Scale–Cognitive Subscale; B-ADL, Bayer Activities of Daily Living; CBF, Cerebral Blood Flow; PC, Posterior Cingulate.

† These four publications derive from the same parent trial (SMART, Trial) and represent complementary rather than independent evidence.

‡ These two publications are companion papers from the same single-group pilot trial.

Because sustained training at these intensities is physiologically demanding for older adults, programs often use periodized or cyclic approaches, with initial mesocycles focusing on hypertrophy to establish a safe foundation, followed by phases targeting maximal strength (Macaulay et al., 2021). Total training volume is generally lower than in moderate-intensity hypertrophy protocols, but intensity is high enough to elicit substantial neuromuscular adaptations.

Considering that high-intensity resistance training may maximize gains in muscle strength, it is also reasonable to investigate its effects on cognitive function, as even small associations may lead to cumulative effects through factors underlying cognitive enhancement (Mavros et al., 2017). These effects reflect increased cortical activation, improved attention, and enhanced neuromuscular engagement, which together may facilitate information processing and reaction speed.

It should be noted that Fiatarone Singh et al. (2014), Suo et al. (2016), Mavros et al. (2017), and Broadhouse et al. (2020) all derive from the same randomized controlled trial–the SMART Trial (n = 100 older adults with MCI) – and report distinct outcome domains from the same cohort. These publications are therefore interpreted as complementary evidence from a single independent trial rather than independent replications. Similarly, Macaulay et al. (2021) and Macaulay et al. (2022) are companion publications from the same single-group pilot trial (n = 20), reporting cognitive and neuroimaging outcomes respectively from identical participants.

Longer-term interventions provide evidence of more sustained cognitive and structural brain benefits. Fiatarone Singh et al. (2014) reported that 10 weeks of high-intensity resistance training in older adults with mild cognitive impairment (n = 100, aged 55–89) improved global cognitive function, executive function, and verbal memory, with some effects persisting for up to 12 months post-intervention. Broadhouse et al. (2020) demonstrated that 6 months of progressive high-intensity training improved executive functions and protected hippocampal regions from degeneration for at least 1 year after the intervention, effects not observed with cognitive training alone.

High-intensity resistance training also positively influences structural brain changes. Best et al. (2015) examined 155 women aged 65–75 over a 52-week intervention and observed improvements in executive function and verbal memory, with slowed white matter atrophy sustained at a 2-year follow-up. Bolandzadeh et al. (2015) similarly reported that twice-weekly high-intensity training over a period of 52 weeks reduced white matter lesion volume in older women, indicating potential long-term neuroprotective effects. Suo et al. (2016) demonstrated that 6 months of high-intensity resistance training increased gray matter volume in the posterior cingulate cortex and reduced white matter lesions in older adults with mild cognitive impairment, with these structural changes linked to improvements in global cognition.

Notably, positive outcomes were observed in both cognitively healthy older adults (Best et al., 2015; Bolandzadeh et al., 2015; Macaulay et al., 2021; Macaulay et al., 2022) and individuals with mild cognitive impairment (Fiatarone Singh et al., 2014; Suo et al., 2016; Broadhouse et al., 2020). While the neuroprotective effects reported in cognitively impaired populations may be particularly clinically relevant, the overall direction of findings was remarkably similar across groups, with improvements observed in executive function, memory, and structural brain health. These results suggest that even in the presence of established cognitive impairment, high-intensity resistance training may remain capable of inducing meaningful cognitive and neurophysiological adaptations.

Mechanistically, high-intensity resistance training may enhance cognitive function both directly, through neuromuscular and cortical activation, and indirectly, via gains in muscular strength. Mavros et al. (2017) found that improvements in muscle strength, rather than aerobic capacity, mediated cognitive gains, supporting the link between strength development and executive function in older adults. Additionally, neurochemical markers, including BDNF and other growth factors, appear to increase following high-intensity training, potentially supporting synaptic plasticity and neural transmission (Sheoran et al., 2023).

High-intensity training can also provide multi-dimensional benefits beyond cognition. Macaulay et al. (Macaulay et al., 2021; Macaulay et al., 2022), reporting cognitive and neuroimaging outcomes from the same pilot trial (n = 20), demonstrated that progressive high-intensity programs improved executive function, working memory, attention, muscular strength, anaerobic capacity, body composition, sleep quality, and overall quality of life in older adults. These studies used autoregulatory progression, allowing participants to adjust load based on performance to ensure safety, illustrating that individualized high-intensity programs are feasible and effective for this population.

While high-intensity resistance training is highly effective, combining it with mentally demanding cognitive interventions may attenuate cognitive benefits, possibly due to excessive mental and physical stress or reduced engagement in everyday activities (Fiatarone Singh et al., 2014; Suo et al., 2016). Therefore, progressive, focused high-intensity resistance training appears beneficial for improving cognitive and neuroprotective outcomes in both cognitively healthy older adults and those with mild cognitive impairment.

## 4 Discussion

Across all training intensities, the direction of findings was broadly similar in cognitively healthy older adults and in those with mild cognitive impairment or dementia. Although study populations, intervention characteristics, and outcome measures varied considerably, resistance training was generally associated with improvements in cognitive performance or attenuation of cognitive decline regardless of baseline cognitive status. These findings suggest that cognitive impairment does not eliminate the potential for cognitive adaptation in response to resistance training and that both healthy and cognitively vulnerable older adults may benefit from appropriately prescribed resistance exercise.

This narrative review synthesised evidence on the effects of resistance training of varying intensity on cognitive function and neurophysiology in older adults. Overall, the findings indicate that resistance training across a broad intensity spectrum–low, moderate, and high–can improve cognitive performance and neurophysiological markers, although the magnitude, mechanisms, and durability of these effects appear to differ by training intensity, volume, and population characteristics.

Across intensity levels, several interrelated mechanisms may underpin the observed cognitive benefits. Resistance training has been associated with increased cerebral blood flow, enhanced expression of neurotrophic factors such as brain-derived neurotrophic factor (BDNF), insulin-like growth factor-1 (IGF-1), and vascular endothelial growth factor (VEGF), reductions in systemic inflammation, and improvements in insulin sensitivity. Structural adaptations, including reduced white matter lesion progression and preservation of hippocampal volume, may further contribute to maintained cognitive function. In addition, resistance training may exert indirect cognitive effects through improvements in physical fitness, functional independence, mood regulation, and sleep quality–factors that are themselves closely linked to cognitive health in older adults.

### 4.1 Low-intensity resistance training and cognitive function

Low-intensity resistance (muscular endurance) training demonstrated modest but consistent benefits for cognitive outcomes, particularly in older adults with cognitive impairment or low baseline physical fitness. Improvements were observed in global cognition, working memory, executive functions, EEG parameters, and neurotrophic and inflammatory biomarkers (e.g., BDNF and IL-8). These effects may be mediated through enhanced cerebral blood flow, increased cardiovascular response, and neuromuscular engagement, especially when exercises are performed at a faster tempo.

However, the evidence base for low-intensity training is limited by a lack of direct comparisons with higher-intensity protocols and by methodological heterogeneity across studies. Many investigations (Yoon et al., 2017; Yoon et al., 2018; Hong et al., 2018; Sardeli et al., 2018) used elastic-band protocols and included non-exercising or stretching control groups, making it difficult to determine whether low-intensity resistance training is superior or merely beneficial relative to inactivity. Furthermore, improvements in body composition together with strength were absent in 7 of the 9 low-intensity studies, suggesting that low-intensity training may be insufficient to induce broader physiological adaptations that could support long-term cognitive health and raising questions about whether the observed cognitive benefits reflect a true training effect or are attributable to other factors such as social engagement or increased physical activity relative to sedentary controls. Nevertheless, low-intensity resistance training remains clinically relevant due to its accessibility, safety, and feasibility for frail or cognitively impaired older adults. It may serve as an entry point to structured exercise or as a complementary modality for individuals unable or unwilling to engage in higher-intensity training.

### 4.2 Moderate-intensity resistance training and its dual benefits for physical and cognitive health

Moderate-intensity resistance training aimed at muscular hypertrophy (approximately 65%–79% of 1RM) consistently improved cognitive function across multiple domains, including attention, reaction time, working memory, and executive function. Both acute and chronic interventions demonstrated beneficial effects, with evidence suggesting an inverted-U relationship between exercise intensity and cognitive performance, whereby moderate intensity may provide optimal arousal and neurochemical responses, e.g., in dopamine, norepinephrine, BDNF (Hsieh et al., 2016).

Several studies (Smolarek et al., 2016; Chen et al., 2017) reported concurrent improvements in muscle strength, body composition, and functional capacity, supporting the notion that moderate-intensity resistance training offers dual benefits for physical and cognitive health. Acute improvements in cognition were also observed following single sessions (Hsieh et al., 2016; Johnson et al., 2016; Tsai et al., 2017; Sardeli et al., 2018; Tsai et al., 2019; Arazi et al., 2021), likely mediated by transient increases in arousal and growth factors such as IGF-1 and BDNF. Longitudinal interventions (Best et al., 2015; Gomes de Souza Vale and Ferreira, 2014; Bolandzadeh et al., 2015; Hsieh et al., 2016; Tsai et al., 2015) further suggested that repeated moderate-intensity training may slow cognitive decline and enhance information processing speed, although the persistence of these effects after training cessation remains insufficiently studied.

Moderate-intensity resistance training appears to be practical and sustainable for older adults, making it a promising candidate for public health interventions. However, it should be noted that many studies in this category compared resistance training against aerobic training or non-exercising controls rather than against other resistance training intensities directly, which limits the strength of conclusions about its relative superiority. Variability in training protocols, cognitive assessment tools, and participant characteristics further limits direct comparisons across studies and underscores the need for standardised methodological frameworks.

### 4.3 High-intensity resistance training and neuroprotective effects

High-intensity resistance training (≥80% 1RM) shows strong evidence for neuroprotective effects, including improvements in global cognition, executive function, working memory, attention, and verbal memory, alongside preservation of structural brain integrity. Several independent studies (Best et al., 2015; Bolandzadeh et al., 2015) report reductions in white matter lesion volume, a finding also observed across multiple publications from the SMART Trial (Suo et al., 2016; Broadhouse et al., 2020), which should collectively be considered a single independent source of evidence. Additionally, there were reports of preservation of hippocampal regions (Broadhouse et al., 2020), and sustained cognitive benefits up to one or 2 years post-intervention, suggesting long-lasting neuroplastic adaptations. High-intensity training appears effective in both healthy older adults and individuals with mild cognitive impairment, although it is noted that a substantial portion of this evidence derives from the SMART Trial and its companion publications, which share the same participant cohort and should be weighted accordingly.

The cognitive benefits of high-intensity training appear to be mediated primarily by gains in muscle strength rather than aerobic capacity, highlighting the importance of neuromuscular adaptations in cognitive ageing. When appropriately periodized and individually tailored, it can provide robust cognitive, neuromuscular, and overall health benefits, making it a promising intervention for mitigating age-related cognitive decline. However, high-intensity protocols require close supervision, appropriate progression, and careful consideration of individual health status. Additionally, some evidence (Hsieh et al., 2016; Suo et al., 2016) suggests that combining intensive resistance training with demanding cognitive training may attenuate cognitive benefits, possibly due to excessive stress or reduced engagement in other cognitively stimulating activities.

### 4.4 Evidence supporting moderate-intensity resistance training as a promising approach

Several studies included in this narrative review notably suggest that moderate-intensity resistance training may produce the most consistent and robust cognitive benefits, emphasizing its potential as a simple and sustainable intervention. It should be acknowledged, however, that this conclusion rests largely on indirect rather than head-to-head evidence: most studies grouped under moderate intensity compared resistance training against aerobic training or non-exercising controls, rather than against low- or high-intensity resistance training protocols. The few studies that did directly compare intensity levels–notably (Naderi et al., 2019) and (Coelho-Júnior et al., 2020) – consistently favoured moderate intensity, lending support to this pattern, but the direct comparative evidence base remains limited. Moderate intensity therefore appears promising rather than established as definitively optimal, and this distinction should be kept in mind when interpreting the findings below.

Cognitive benefits may become evident even after a single training session (Hsieh et al., 2016; Tsai et al., 2017; Arazi et al., 2021). Following sustained training programs, these effects can persist for up to 1.5 years after the cessation of training (de Almeida et al., 2021; Sheoran et al., 2023), with consistent training increasing both the magnitude and duration of cognitive benefits (Naderi et al., 2019). Sessions lasting 30–60 min and engaging the body’s large muscle groups are considered most effective.

One possible explanation lies in the balance between physiological stimulation and systemic stress. Moderate intensity may provide sufficient metabolic and neuromuscular challenge to stimulate neurotrophic factor release and promote neuroplastic adaptations, while avoiding excessive physiological strain that could elevate cortisol levels or induce prolonged fatigue. From the perspective of the inverted-U model of arousal, moderate intensity may optimize acute cognitive performance by inducing an optimal level of neurophysiological activation, whereas very low intensities may fail to sufficiently stimulate adaptive processes and very high intensities may temporarily impair cognitive performance due to excessive stress responses (Hsieh et al., 2016). Furthermore, moderate-intensity training may allow for higher adherence and better recovery in older populations, enabling more consistent long-term exposure to beneficial stimuli. Chronic adaptations likely depend on cumulative neuroplastic changes, and a sustainable training stimulus may therefore be more important than maximal loading. In contrast, high-intensity protocols, while potentially potent in stimulating anabolic and neurotrophic pathways, may not be tolerated equally well by all older adults, particularly those with comorbidities or lower baseline fitness levels. Although positive effects are observed across all intensity levels, moderate intensity may therefore be regarded as particularly promising for maintaining cognitive function in older adults, a conclusion most directly supported by (Naderi et al., 2019) and (Coelho-Júnior et al., 2020), the two studies offering direct intensity comparisons within the included literature.

### 4.5 Methodological limitations and future directions

Despite promising findings, several limitations must be acknowledged. The included studies varied substantially in sample size, ranging from very small samples (e.g., n = 11 in (Hsieh et al., 2016)) to larger samples (e.g., n = 155 in Best et al. (Best et al., 2015)). Many studies lack sufficient statistical power, particularly for detecting differential cognitive effects across intensity levels. Small samples are more vulnerable to imprecision and unstable effect estimates, and positive findings from small samples may be overrepresented due to publication bias, potentially inflating the overall picture of benefits. At the same time, where small-sample findings converge with those of larger trials, this consistency lends additional credibility to the observed effects. Cognitive assessment tools also varied widely, limiting comparability across studies. Few investigations included long-term follow-up, making it difficult to determine the persistence of cognitive benefits.

A further methodological limitation concerns the assumed equivalence of intensity across resistance training modalities. Several included studies–particularly in the low-intensity category–used elastic-band protocols (Hong et al., 2018; Yoon et al., 2017; Yoon et al., 2018; Urzi et al., 2019), while moderate- and high-intensity studies predominantly employed free weights or machines. Although intensity was expressed in comparable relative terms across studies, the absolute mechanical stimulus, loading profile, neuromuscular demand, and potential for progressive overload differ substantially between modalities, and these differences may influence motor unit recruitment, time under tension, and ultimately the neurophysiological adaptations underlying cognitive benefits.

This raises the possibility that absolute mechanical load, rather than relative intensity alone, may be a critical determinant of neurophysiological adaptation–and that only modalities capable of generating sufficient mechanical stimulus, such as free weights or machines, can reliably elicit the neurotrophic responses most strongly associated with cognitive enhancement. Elastic-band protocols may not consistently reach this threshold, potentially explaining the comparatively modest cognitive gains observed in elastic-band-dominated studies. Future research should therefore treat modality as an independent variable when examining intensity-related effects on cognition.

Future research should further focus on large-scale, randomized controlled trials that directly compare resistance training intensities while carefully controlling for optimal volume, frequency, and exercise selection. Additionally, studies should investigate the long-term persistence of effects and explore how resistance training interacts with other forms of exercise to maximize cognitive outcomes. Longitudinal studies with extended follow-up periods are needed to determine whether resistance training can delay the onset of dementia or other neurodegenerative conditions. Additionally, mechanistic studies integrating neuroimaging, biomarkers, and behavioural outcomes would clarify the pathways through which resistance training at different intensities influences cognitive aging.

However, since several positive effects persist even for a longer period after training has stopped, older adults should be encouraged to engage in moderately intense resistance training in order to reduce brain aging-related changes and maintain cognitive function in the long term.

### 4.6 Practical implications

Collectively, the evidence suggests that resistance training is a promising non-pharmacological intervention for preserving cognitive health in older adults. Moderate-intensity resistance training appears to offer a favourable balance between feasibility and efficacy, while high-intensity training may provide superior neuroprotective benefits when safely implemented. Low-intensity training remains valuable for vulnerable populations and as a gateway to more demanding exercise modalities.

From a clinical and public health perspective, older adults should be encouraged to engage in regular, progressive resistance training tailored to individual capacity, with the goal of maximising muscle strength while ensuring safety. Such interventions may contribute not only to improved physical function but also to the maintenance of cognitive performance and quality of life during aging.
